# Sex-associated differences in excitability within the bed nucleus of the stria terminalis are reflective of cell-type

**DOI:** 10.1016/j.ynstr.2018.100143

**Published:** 2018-12-19

**Authors:** Hannah E. Smithers, John R. Terry, Jonathan T. Brown, Andrew D. Randall

**Affiliations:** a Institute of Biomedical and Clinical Sciences, University of Exeter Medical School, Hatherly Laboratory, Exeter, EX4 4PS, UK; b College of Engineering, Mathematics and Physical Sciences, University of Exeter, Living Systems Institute, EX4 4QD, UK

## Abstract

The bed nucleus of the stria terminalis (BNST) is a sexually dimorphic brain region which plays a key role in stress, anxiety, and anxiety-related disorders. Human females have an increased susceptibility to anxiety-related disorders, however the physiological basis of this is not fully understood. Here we examined the effect of the oestrous cycle and sex on the electrophysiological properties of Type I and Type II cells in the anterolateral area of the BNST (BNST^ALG^) in unstressed animals. There was no significant effect of oestrous cycle on any of the parameters examined in either cell type. Compared to males, the female cohort had lower capacitance in Type I cells while having a higher capacitance in Type II cells. Type II cells also displayed decreased excitability in the female cohort. In order to confirm the effect of these populations on stress and anxiety, a correlation with behaviour on the elevated zero maze was carried out. We observed that increased excitability in Type II neurons correlated with a decrease in anxiety-like behaviour. These sex-specific differences in excitability may contribute to altered susceptibility to anxiety-related disorders.

## 1 Introduction

Chronic stress and anxiety are placing increasing burdens on the economies of western societies (Kessler et al., 2001). A number of the detrimental effects of chronic stress are mediated via the hypothalamic-pituitary-adrenal (HPA) axis originating from the paraventricular nucleus (PVN). A key source of stress-related responses is the limbic system however there are no extensive connections between higher limbic system structures, such as the hippocampus or the medial prefrontal cortex, and the PVN. Instead connections are relayed through intermediate brain regions, a key one of which is the bed nucleus of the stria terminalis (BNST).

Classically, the BNST has been hypothesised to mediate “sustained” fear and/or anxiety after repeated stressors, as well as contextual cues that predict aversive and/or stressful stimuli (Davis et al., 2010) however a number of more recent studies have highlighted the central role of the BNST in learned fear (Moaddab and Dabrowska, 2017; Janeček and Dabrowska, 2018), indicating that the BNST and the amygdala work together to process fearful stimuli (Shackman and Fox, 2016). Due to the central role of the BNST in stress processing it has been implicated in a number of anxiety-related disorders including general anxiety disorder (GAD) (Pigott, 2003; Conrad et al., 2011; Yassa et al., 2012), drug addiction (specifically stress-induced relapse) (Shaham et al., 2003; Dumont et al., 2005; Li et al., 2012; Jennings et al., 2013) and post-traumatic stress disorder (PTSD) (Hammack et al., 2012; Lebow et al., 2012; Janitzky et al., 2015; Rodriguez-Sierra et al., 2016).

Anxiety-related disorders show a significantly higher rate of incidence in females relative to males, with some estimates putting rates of PTSD in females at twice that observed in males (Kessler et al., 1995; Breslau and Kessler, 2001). While societal effects may contribute to such elevated rates, alterations in underlying neurological stress processing may also be involved. In this regard it is important to note that the BNST is a sexually dimorphic brain region in both humans and rodents (del Abril et al., 1987; Malsbury and McKay, 1987; Hines et al., 1992; Wittmann and McLennan, 2013). Moreover, it plays a role in sexual identity (Hines et al., 1992; Kruijver et al., 2000) and sex-specific behaviours. These behaviours include maternal behaviour (Klampfl et al., 2014, 2016), aggression in males (Trainor et al., 2010; Masugi-Tokita et al., 2016), sexual behaviour (He et al., 2013; Maejima et al., 2015) and sex-specific responses to stressors (Greenberg et al., 2014; Janitzky et al., 2014).

The BNST is comprised of a number of anatomically defined subregions (Dong et al., 2001b, 2001a). One such subdivision is the anterolateral area of the BNST (BNST^ALG^), a region with extensive connections to the PVN that has been strongly implicated in anxiety-related response (Hammack et al., 2007; Daniel and Rainnie, 2015). There are three neurophysiologically distinct cell types in the BNST^ALG^ which were first described by Hammack et al. (2007). While the physiological roles of these cell types have yet to be fully determined, a recent review of existing data hypothesised the role that each of these cell types fulfil. The exact role of Type I and Type II cells are yet to be fully understood however they are believed to represent ‘anxiety off’ cells while Type III cells, which are predominantly CRF positive-cells, are ‘anxiety on’ cells (Daniel and Rainnie, 2015).

A number of studies have found an effect of oestrous cycle on the electrophysiological properties of neurons (Blume et al., 2017; Vandegrift et al., 2017; Proano et al., 2018). In order to determine if sex specific differences were being driven by oestrous cycle the electrophysiological properties were compared at each stage of the cycle. As no statistically significant differences were observed the females at each stage were grouped together for comparison with age matched males from the same colony. We hypothesised that sex-specific differences in the intrinsic properties of neurons located in the anterolateral area of the BNST may be contributing to sex-associated differences in stress processing. We sought to examine this by combining neurophysiological characterizations of BNST cellular populations with behavioural assessments of anxiety made using the elevated zero maze.

## 2 Method

### 2.1 Animals

All experiments were carried out in accordance with the Animals (Scientific Procedures) act 1986. All tissues for this study were harvested from C57-BL6 mice bred in house from initial stock purchased from Charles River. For their entire lifespan animals had *ad libitum* access to both food and water and were housed on a 12/12 light-dark cycle. In this investigation male and female animals aged 3–5 months were compared; the female cohort had an age range of 15–26 weeks with a mean age of 21 weeks while the male cohort was recorded from animals aged 14–23 weeks with a mean age of 18 weeks. The female cohort was further divided based on their stage in the oestrous cycle. Experimental days employing brain slices obtained from the two different sexes and various oestrous stages were interleaved throughout the duration of the study. A proportion of these animals underwent behavioural tests. On the days in which behaviour was employed, the zero maze was run in the morning and the animal was sacrificed for subsequent slicing within an hour of the maze.

### 2.2 Behavioural tests

Anxiety levels were examined using the elevated zero maze (Faria et al., 2016). In this maze, anxiety is thought to be related to the proportion of time spent in the open area. A light source was placed in the centre of the maze which allowed the two ‘closed’ quadrants of the maze to remain dark while the ‘open’ quadrants of the maze were illuminated by white light. Animals were not handled by the experimenter prior to behavioural tests. Animals were placed in the maze for 5 min and the proportion of time spent in the open vs closed component was determined using a stop watch. The experimenter was blind by to animal's sex and oestrous cycle stage during analysis.

### 2.3 Slice preparation

Animals were killed by cervical dislocation, the skull was opened and the brain was rapidly removed and placed immediately in an ice-cold slicing medium consisting of (in mM): 189 Sucrose, 10 D-Glucose, 26 NaHCO_3_, 3 KCl, 5 MgSO_4_, 0.1 CaCl_2_, 1.25 NaH_2_PO_4_. A Leica VT1200 vibratome was then used to cut serial 300 μm thick coronal sections. Following their preparation slices were allowed to recover at room temperature for at least 60 min in our standard artificial cerebrospinal fluid (aCSF). This was composed of (in mM):124 NaCl, 3 KCl, 24 NaHCO_3_, 1.25 NaH_2_PO_4_, 2 CaCl_2_, 1 MgSO_4_, 10 D-Glucose, and was continuously gassed with carbogen (i.e. 95%O_2_,5%CO_2_)

Slices containing the BNST^ALG^ came from approximately Bregma -.1 to +0.3, and were identified with the aid of the Paxinos and Franklin mouse brain atlas using the anterior commissure as a key landmark. Recordings were carried out in the dorsal portion of the BNST^ALG^. Typically one or two suitable BNST-containing coronal sections per animal could be used and by bisecting these along the dorsal-ventral midline we were able to obtain two to four usable tissue sections per mouse.

### 2.4 Oestrous cycle testing

Following the cervical dislocation of female mice and the removal of the brain into ice cold sucrose solution the stage of oestrous was examined via a wet smear. 10 μL of PBS was inserted ∼ 5 mm into the mouse's vagina and gently flushed two to three times. The final flush was collected and placed on to a slide for visual examination. The unstained sample was examined under a ×10 objective on a Nikon eclipse E800 microscope. Oestrous cycle stage was determined by the proportion of each cell type present as described in Caligioni (2009). Briefly, during proestrus the sample is predominantly populated with nucleated epithelial cells, during estrus it is predominantly anucleated cornified cells, in metestrus it is a combination of leukocytes, cornified and nucleated epithelial cells and during diestrus it is predominantly leucocytes.

### 2.5 Electrophysiological recordings

All recordings were made using the whole cell patch clamp technique. The BNST containing brain slice was transferred into a submerged recording chamber which was perfused with gassed aCSF and maintained at a temperature of ∼34.5 °C. The recording chamber was mounted on the stage of an upright microscope (Olympus BX51).

A Flaming Browning P-97 micropipette puller was used to produce the microelectrodes used in this study. These had a resistance of 3–5 MΩ when filled with the K-Gluconate-based internal solution used for all recordings. This was composed of (in mM): 130 K-Gluconate, 20 KCl, 10 HEPES free acid, 0.2 EGTA, 0.3 GTP-Na salt, ATP-Mg salt, pH adjusted to 7.3 with KOH. The 15 mV junction potential error produced by pairing this pipette solution with our aCSF was corrected for during analysis.

Cells within the BNST were visually identified using the microscope's infrared differential interference contrast optics and a coupled IR-sensitive CMOS camera (Thor Labs). All recordings were made with a Multiclamp 700B amplifier (Molecular Devices) interfaced to a Digidata 1440A (Molecular Devices). Experiments were controlled and data collected using the Clampex programme within the pClamp 10.4 software suite. All data were stored directly onto a personal computer (Hewlett-Packard) and backed-up to a network drive. A series of electrophysiological protocols were carried out as previously described in Smithers et al. (2017). Cells were then sorted into their subtypes based on their electrophysiological properties from two different prestimulus potentials based on criteria outlined in Hammack et al. (2007).

### 2.6 Data analysis

Data were analysed using a range of custom written MATLAB 2014b scripts and pClamp 10.4 software. Determination of appropriate statistical test was based on assessment of normal distribution using the Shapiro-Wilk normality test. Parameters were measured either at rest or from two preset prestimulus potentials of −70 mV or −80 mV.

#### 2.6.1 Statistical analysis

For comparisons of properties which were only examined at one potential, a one-way ANOVA was used to examine oestrous and an unpaired *t*-test was used to examine sex for populations determined to be normally distributed. For populations which were not normally distributed a Kruskal-Wallis test was performed to examine oestrous and a Mann-whitney *U* test was performed to examine sex. For properties which were examined from two prestimulus potentials, if the data were normally distributed at both prestimulus membrane potentials a repeated measure two-way ANOVA was carried out with oestrous or sex as the between subject effect and prestimulus membrane potential as the within subject effect. If the data was not normally distributed at one or both of the prestimulus potentials a Kruskal-Wallis test or Mann-whitney U was performed at each prestimulus membrane potential as appropriate. Firing frequencies in response to each depolarising current injection were analysed via repeated measure three-way ANOVA. All the above statistical tests were carried out in SPSS. Proportion of cells firing was determined by a chi squared test carried out in Excel. Figures were prepared with Origin 2016.

## 3 Results

### 3.1 Behaviour

**Fig. 1:**
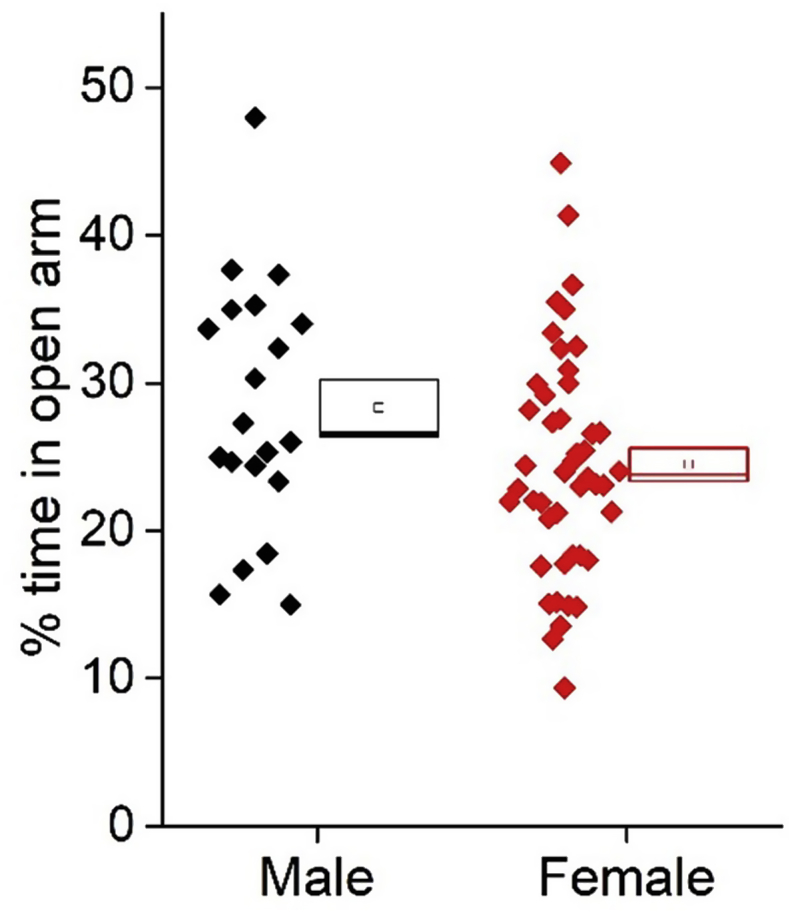
Percentage time spent in the open of the elevated zero maze of each sex. (Male n = 20, female, n = 46).

Anxiety levels were assessed using the elevated zero maze, where mice were run on the maze in the early hours of their lights-on stage. Anxiety was measured as the percentage of time the animals spent in the light component of the maze. While the females spent approximately 4% less time in the open in comparison to the males (male: 28 ± 2%, n = 20, female: 24 ± 1%, n = 46) this was not statistically significant (unpaired *t*-test, p = 0.07, degrees of freedom (df) = 64, Fig. 1). There was no statistically significant effect of oestrous cycle on the % time spent in the open component of the maze (Table 1).

**Table 1:** % time spend in the open of each cohort on the elevated zero maze, df = 45.

	Diestrus	Proestrus	Estrus	Metestrus	P value	Statistical test
% time in the open arm	21 ± 2 (n = 12)	28 ± 3 (n = 10)	23 ± 2 (n = 13)	27 ± 2 (n = 11)	0.07	One-way ANOVA

### 3.2 Electrophysiological properties of neurons located in the anterolateral area of the BNST

Cells located in the BNST^ALG^ were sorted based on their electrophysiological properties based on criteria outlined in Hammack et al. (2007). Type I and Type II neurons both display a sag following the injection of a depolarising current injection indicative of I_h_ current. Type I neurons have a regular spiking pattern while Type II neurons exhibit a burst firing pattern in response to depolarising stimuli. Type II neurons also exhibit a rebound depolarisation upon termination of a hyperpolarising current which often reached threshold from a holding potential of −55 mV. A total of 79 neurons were recorded from BNST^ALG^ neurons from male mice and 147 from female mice. In the male cohort a total of 21 Type I cells, 30 Type II cells, 11 Type III cells and 17 cells which did not fit the criteria of the 3 populations were recorded. In the female cohort a total of 66 Type I cells, 56 Type II cells, 10 Type III cells and 15 cells which did not fit the criteria were recorded. To determine if there is a relationship between oestrous cycle and the intrinsic electrophysiological properties of these neurons, the stage of oestrous was determined at the point of death.

#### 3.2.1 Type I neurons

Of the 66 Type I cells recorded from the female cohort, 13 were from mice in estrus, 16 were from mice in metestrus, 21 were from mice in diestrus and 16 were from mice in proestrus. There were no statistically significant effects of the different stages of oestrous on the resting membrane potential (Table 2), passive membrane properties (Table 3) or action potential properties (Table 4) of Type I neurons located in the BNST^ALG^. There was also no effect of the oestrous cycle on the proportion of cells firing in response to depolarising current injections. The firing frequencies of Type I neurons was also not dependent upon oestrous stage (repeated measure two-way ANOVA, p = 0.6, df = 3).

**Table 2:** Resting membrane potential (RMP) and firing frequency at resting membrane potential of Type I neurons in oestrous cycle. Degrees of freedom for between group comparisons = 3.

Parameter	Estrus	Metestrus	Diestrus	Proestrus	P value	Statistical test
RMP (mV)	−64 ± 4 (n = 13)	−70 ± 3 (n = 16)	−68 ± 2 (n = 21)	−71 ± 2 (n = 18)	0.28	One-way ANOVA
Frequency at RMP (Hz)	2.1 ± 0.7 (n = 8)	5.4 ± 2.1 (n = 7)	8.6 ± 2.9 (n = 9)	4.1 ± 2.1 (n = 7)	0.19	One-way ANOVA

**Table 3:** Passive membrane properties of Type I neurons in oestrous cycle. Kruskal-Wallis test (KW) statistical test. Degrees of freedom for between group comparisons = 3.

Parameter and prestimulus potential		Estrus	Metestrus	Diestrus	Proestrus	P value	Statistical test
R_in_ (MΩ)	−70 mV	510 ± 46 (n = 13)	536 ± 59 (n = 16)	473 ± 43 (n = 21)	535 ± 44 (n = 18)	0.63	KW
	−80 mV	427 ± 49 (n = 13)	446 ± 61 (n = 16)	391 ± 39 (n = 21)	444 ± 44 (n = 18)	0.80	KW
Tau (ms)	−70 mV	28 ± 3 (n = 13)	35 ± 3 (n = 16)	31 ± 4 (n = 21)	31 ± 3 (n = 18)	0.51	Two-way ANOVA
	−80 mV	22 ± 2 (n = 13)	27 ± 3 (n = 16)	25 ± 3 (n = 21)	28 ± 3 (n = 18)		
Sag (%)	−70 mV	7 ± 1 (n = 13)	9 ± 1 (n = 16)	6 ± 2 (n = 21)	8 ± 1 (n = 18)	0.52	KW
	−80 mV	14 ± 3 (n = 13)	15 ± 1 (n = 16)	16 ± 1 (n = 21)	16 ± 3 (n = 18)	0.67	KW
Capacitance (pF)	−70 mV	56 ± 5 (n = 13)	70 ± 6 (n = 16)	67 ± 7 (n = 21)	62 ± 6 (n = 18)	0.51	KW
	−80 mV	57 ± 6 (n = 13)	66 ± 6 (n = 16)	66 ± 5 (n = 21)	68 ± 7 (n = 18)	0.76	KW

**Table 4:** Action potential properties of Type I neurons in oestrous cycle. Kruskal-Wallis test (KW) statistical test. Degrees of freedom for between subject effects are 3.

Parameter and prestimulus potential		Estrus	Metestrus	Diestrus	Proestrus	P	Statistical test
Zenith (mV)	−70 mV	18 ± 2 (n = 13)	17 ± 2 (n = 16)	15 ± 2 (n = 21)	13 ± 2 (n = 18)	0.30	KW
	−80 mV	20 ± 2 (n = 13)	17 ± 2 (n = 16)	17 ± 2 (n = 21)	15 ± 2 (n = 18)	0.46	KW
Width (ms)	−70 mV	0.75 ± 0.03 (n = 13)	0.75 ± 0.04 (n = 16)	0.73 ± 0.03 (n = 21)	0.77 ± 0.03 (n = 18)	0.47	KW
	−80 mV	0.73 ± 0.03 (n = 13)	0.74 ± 0.05 (n = 16)	0.72 ± 0.04 (n = 21)	0.75 ± 0.04 (n = 18)	0.74	KW
Threshold (mV)	−70 mV	−51 ± 2 (n = 13)	−51 ± 1 (n = 16)	−52 ± 1 (n = 21)	−51 ± 1 (n = 18)	0.81	Two-way ANOVA
	−80 mV	−50 ± 2 (n = 13)	−51 ± 1 (n = 16)	−52 ± 1 (n = 21)	−51 ± 1 (n = 18)		
dV/dt max (mV/ms)	−70 mV	272 ± 23 (n = 13)	281 ± 16 (n = 16)	265 ± 16 (n = 21)	233 ± 11 (n = 18)	0.14	KW
	−80 mV	308 ± 24 (n = 13)	302 ± 20 (n = 16)	294 ± 17 (n = 21)	257 ± 11 (n = 18)	0.17	KW

As no statistically significant effects of oestrous cycle was observed within this population the cells from each stage of oestrous were grouped together to form the female cohort. This female cohort was then compared with an age-matched male cohort to examine the effect of sex on this population of cells. We began by looking at the resting membrane potential of Type I cells, both cohorts had similar resting membrane potentials of ∼ −69 mV (male: 70 ± 2 mV, n = 26, female: 69 ± 1 mV, n = 68, unpaired *t*-test, p = 0.53, df = 92). Following this we examined the firing properties of cells at their resting membrane potential. There was no effect of sex on the proportion of cells firing with 10/26 male cells firing and 31/68 female cells firing (chi-squared, p = 0.53). We also examined the firing frequency of the cells which were firing at rest and found no sex-dependent effect (Male: 2.2 ± 0.5, n = 10, Female: 5.2 ± 1.1 Hz, Mann-Whitney p = 0.36, n = 31).

**Fig. 2:**
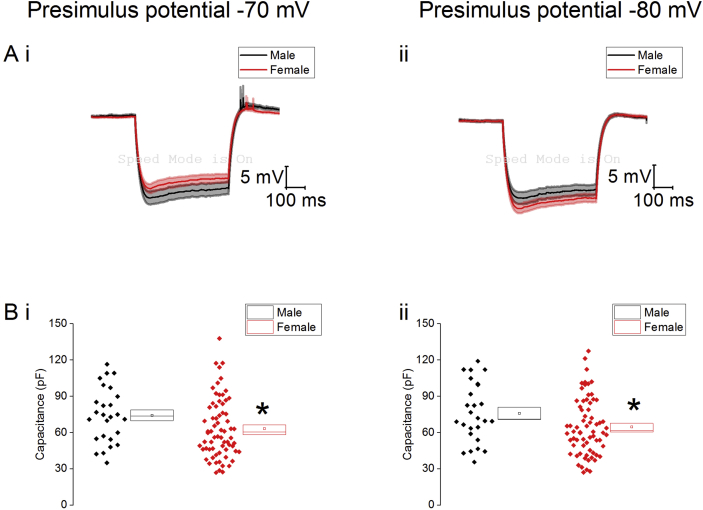
Passive membrane properties of Type I neurons. A) average responses to the injection of −40 pA of current. B) Effect of sex on capacitance of Type I neurons a prestimulus potential of −70 mV (i) and −80 mV (ii), ∗ = p < 0.05.

Due to the voltage-dependent nature of a number of electrophysiological properties cells were held at two prestimulus potentials of −70 mv and −80 mV. We began by examining the passive membrane properties of the neurons in response to the injection of −40 pA of current (average trace shown in Fig. 2). One key parameter measured was the capacitance of the cell which relates to the size of the neuron. In type I neurons the female cohort had a significantly lower capacitance from both prestimulus potentials, (−70 mV: male: 74 ± 4, n = 26, female: 63 ± 3, n = 68, Mann-Whitney U, p = 0.04. −80 mV: male: 76 ± 5, n = 26, female: 65 ± 3, n = 68, Mann-Whitney U, p = 0.04, Fig. 2) indicating smaller Type I cells in females. No differences were found in the any of the other passive membrane properties (Table 5).

**Table 5:** Passive membrane properties of Type I neurons. Mann-Whitney U (MW) statistical test, degrees of freedom in Two-way ANOVA = 1.

Parameter and prestimulus potential		Male	Female	P value	Statistical test
R_in_ (MΩ)	−70 mV	496 ± 37 (n = 26)	511 ± 24 (n = 68)	0.73	MW
	−80 mV	384 ± 28 (n = 26)	425 ± 24 (n = 68)	0.48	MW
Tau (ms)	−70 mV	35 ± 2 (n = 26)	31 ± 2 (n = 68)	0.25	Two-way ANOVA
	−80 mV	27 ± 2 (n = 26)	26 ± 1 (n = 68)		
Sag (%)	−70 mV	8 ± 1 (n = 26)	7 ± 1 (n = 68)	0.9	MW
	−80 mV	14 ± 1 (n = 26)	15 ± 1 (n = 68)	0.8	MW

The action potential properties were examined from the first action potential generated in response to a series of depolarising current injections. There was no effect of sex on action potential zenith, width, threshold or maximum rate of rise (dV/dt max) (Table 6). The excitability of cells was examined using a number of different protocols, these include incremental current steps ranging from 5 pA to 80 pA in 15 pA increments, from this we examine the proportion of cells firing in response to each stimuli, the frequency of firing in each cohort (repeated measure three-way ANOVA, p = 0.64, df = 1) and latency following the injection of 80 pA of current (−70 mV: male: 12 ± 1 ms, n = 26, female: 13 ± 1 ms, n = 68, Mann-Whitney U, p = 0.74. −80 mV: male: 27 ± 3 ms, n = 26, female: 34 ± 5 mV/ms, n = 68, Mann-U-Whitney, p = 0.10) and observed no significant effects.

**Table 6:** Action potential properties of Type I neurons. Mann-Whitney U (MW) statistical test, degrees of freedom in two way ANOVA = 1.

Parameter and prestimulus potential		Male	Female	P	Statistical test
Zenith (mV)	−70 mV	16 ± 2 (n = 26)	15 ± 1 (n = 68)	0.43	MW
	−80 mV	17 ± 2 (n = 26)	17 ± 1 (n = 68)	0.61	MW
Width (ms)	−70 mV	0.76 ± 0.04 (n = 26)	0.75 ± 0.02 (n = 68)	0.99	MW
	−80 mV	0.76 ± 0.04 (n = 26)	0.73 ± 0.02 (n = 68)	0.50	MW
Threshold (mV)	−70 mV	−51 ± 1 (n = 26)	−51 ± 1 (n = 68)	0.69	Two-way ANOVA
	−80 mV	−51 ± 1 (n = 26)	−51 ± 1 (n = 68)		
dV/dt max (mV/ms)	−70 mV	266 ± 12 (n = 26)	262 ± 8 (n = 68)	0.84	Two-way ANOVA
	−80 mV	291 ± 15 (n = 26)	289 ± 9 (n = 68)		

**Fig. 3:**
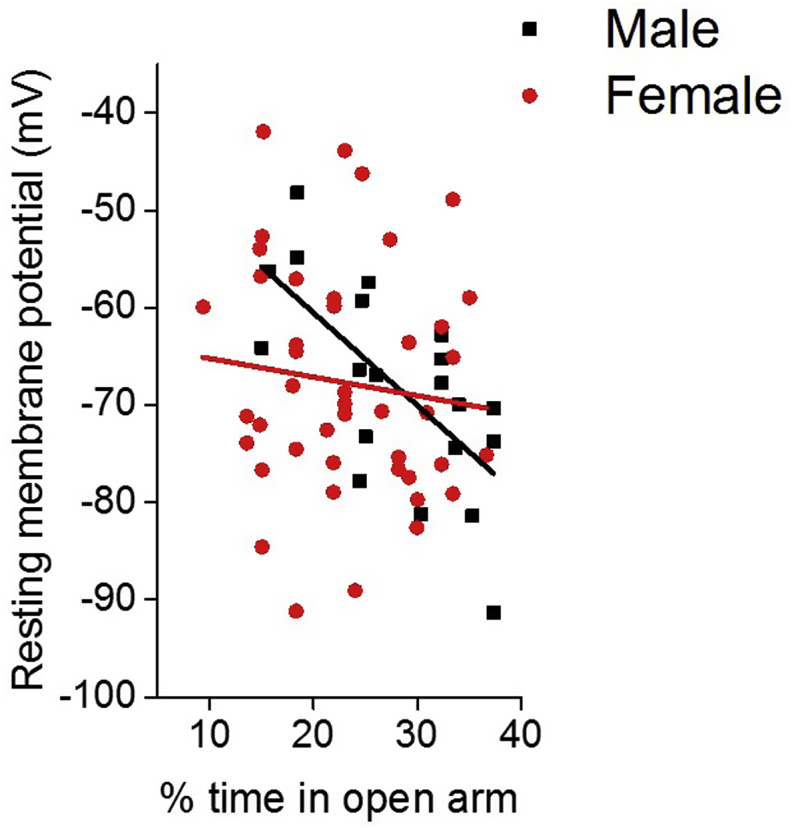
Correlation between the percentage of time spent in the open component of the maze and the resting membrane potential of Type I neurons. Male cohort examined 14 cells taken from 9 animals (ANOVA, p = 0.001), female cohort examined 29 cells taken from 20 animals (ANOVA p = 0.46).

In order to address the role of Type I cells in behaviour the electrophysiological properties described here were correlated with percentage time spent in the open component of the maze. A more depolarised resting membrane potential was highly correlated with decreases in anxiety like-behaviour in Type I neurons, however, interestingly this was only observed in the male cohort (Male: Pearson correlation, R-squared = 0.4, ANOVA p = 0.001, Female: Pearson correlation, R-squared = 0.01, p = 0.46, Fig. 3).

#### 3.2.2 Type II neurons

A total of 30 Type II neurons were recorded from males and 56 Type II neurons were recorded from females. Of the female cells 17 cells were recorded from mice in estrus, 8 cells were recorded from mice in metestrus, 14 cells were recoded from mice in diestrus and 17 cells were recorded from mice in proestrus. No statistically significant effect was observed between any of the groups in resting membrane potential (Table 7), passive membrane properties (Table 8) or action potential properties (Table 9). There was also no effect of oestrous cycle on either the proportion of cells firing in response to each depolarising current injection or the firing frequency of cells in response to a series of depolarising current injections (Repeated measure three-way ANOVA, p = 0.77, df = 3). As no effect of oestrous cycle was detected these cells were grouped together to form the female cohort; this was then compared with an interleaved male cohort from the same colony to examine sex.

**Table 7:** Resting membrane potential of Type II neurons in oestrous cycle. Degrees of freedom for between subject effects are 3.

Parameter	Estrus	Metestrus	Diestrus	Proestrus	P value	Statistical test
RMP (mV)	−68 ± 3 (n = 17)	−68 ± 3 (n = 8)	−69 ± 2 (n = 14)	−69 ± 2 (n = 17)	0.98	One-way ANOVA

**Table 8:** Passive membrane properties of Type II neurons in oestrous cycle. Kruskal-Wallis test (KW) statistical test. Degrees of freedom for between subject effects are 3.

Parameter and prestimulus potential		Estrus	Metestrus	Diestrus	Proestrus	P value	Statistical test
R_in_ (MΩ)	−70 mV	557 ± 66 (n = 17)	412 ± 107 (n = 8)	445 ± 43 (n = 14)	440 ± 44 (n = 17)	0.18	KW
	−80 mV	447 ± 63 (n = 17)	317 ± 90 (n = 8)	346 ± 45 (n = 14)	341 ± 34 (n = 17)	0.25	KW
Tau (ms)	−70 mV	36 ± 3 (n = 17)	29 ± 3 (n = 8)	28 ± 2 (n = 14)	30 ± 3 (n = 17)	0.16	KW
	−80 mV	28 ± 3 (n = 17)	22 ± 4 (n = 8)	23 ± 2 (n = 14)	24 ± 2 (n = 17)	0.30	KW
Sag (%)	−70 mV	11 ± 1 (n = 17)	13 ± 3 (n = 8)	11 ± 1 (n = 14)	12 ± 1 (n = 17)	0.97	KW
	−80 mV	18 ± 2 (n = 17)	21 ± 3 (n = 8)	21 ± 3 (n = 14)	19 ± 2 (n = 17)	0.71	KW
Capacitance (pF)	−70 mV	71 ± 6 (n = 17)	87 ± 14 (n = 8)	66 ± 4 (n = 14)	73 ± 6 (n = 17)	0.52	Repeated measure two-way ANOVA
	−80 mV	71 ± 7 (n = 17)	85 ± 13 (n = 8)	76 ± 7 (n = 14)	73 ± 5 (n = 17)

**Table 9:** Action potential properties of Type II neurons in the stages of the oestrous cycle. Kruskal-Wallis test (KW) statistical test. Degrees of freedom for between subject effects are 3.

Parameter and prestimulus potential		Estrus	Metestrus	Diestrus	Proestrus	P	Statistical test
Zenith (mV)	−70 mV	15 ± 3 (n = 17)	16 ± 2 (n = 8)	10 ± 2 (n = 14)	16 ± 1 (n = 17)	0.14	Repeated measure two-way ANOVA
	−80 mV	17 ± 3 (n = 17)	17 ± 2 (n = 8)	11 ± 2 (n = 14)	17 ± 1 (n = 17)		
Width (ms)	−70 mV	0.80 ± 0.03 (n = 17)	0.72 ± 0.04 (n = 8)	0.74 ± 0.03 (n = 14)	0.75 ± 0.03 (n = 17)	0.40	Repeated measure two-way ANOVA
	−80 mV	0.75 ± 0.04 (n = 17)	0.68 ± 0.03 (n = 8)	0.69 ± 0.03 (n = 14)	0.73 ± 0.03 (n = 17)		
Threshold (mV)	−70 mV	−52 ± 1 (n = 17)	−49 ± 2 (n = 8)	−51 ± 1 (n = 14)	−53 ± 2 (n = 17)	0.52	KW
	−80 mV	−54 ± 1 (n = 17)	−51 ± 2 (n = 8)	−53 ± 1 (n = 14)	−53 ± 1 (n = 17)	0.72	KW
dV/dt max (mV/ms)	−70 mV	238 ± 14 (n = 17)	281 ± 20 (n = 8)	222 ± 13 (n = 14)	258 ± 14 (n = 17)	0.12	Repeated measure two-way ANOVA
	−80 mV	273 ± 15 (n = 17)	313 ± 21 (n = 8)	257 ± 16 (n = 14)	285 ± 14 (n = 17)		

There was no effect of sex on the resting membrane potential of Type II neurons, this averaged −66 ± 2 mV in the males and −68 ± 1 mV in the females (unpaired *t*-test, p = 0.43, df = 84). The proportion of cells firing at rest was also not dependent upon sex with 14/30 male cells firing at rest and 23/56 female cells firing (chi-squared p = 0.62). Of the (40–50% of the total) cells which were firing at rest the average firing frequency was ∼4 Hz in both cohorts (male: 4.9 ± 1.6 Hz, n = 14, female: 4.3 ± 0.8 Hz, Mann-Whitney U, p = 0.96). Action potential properties were examined from the first action potential generated in response to a series of depolarising current injections from two prestimulus potentials. Action potential zenith, width, and maximum rate of rise were not dependent upon sex (Table 10).

**Table 10:** Action potential properties of Type I neurons. Mann-Whitney U (MW) statistical test, degrees of freedom in two way ANOVA = 1.

Parameter and prestimulus potential		Male	Female	P	Statistical test
Zenith (mV)	−70 mV	13 ± 2 (n = 30)	14 ± 1 (n = 56)	0.61	Two-way ANOVA
	−80 mV	14 ± 2 (n = 30)	15 ± 1 (n = 56)		
Width (ms)	−70 mV	0.73 ± 0.02 (n = 30)	0.76 ± 0.02 (n = 56)	0.39	Two-way ANOVA
	−80 mV	0.69 ± 0.03 (n = 30)	0.72 ± 0.02 (n = 56)		
Threshold (mV)	−70 mV	−51 ± 1 (n = 30)	−52 ± 1 (n = 56)	0.52	MW
	−80 mV	−53 ± 1 (n = 30)	−52 ± 1 (n = 56)	0.73	MW
dV/dt max (mV/ms)	−70 mV	250 ± 15 (n = 30)	246 ± 8 (n = 56)	0.92	Two-way ANOVA
	−80 mV	277 ± 16 (n = 30)	278 ± 8 (n = 56)		

**Fig. 4:**
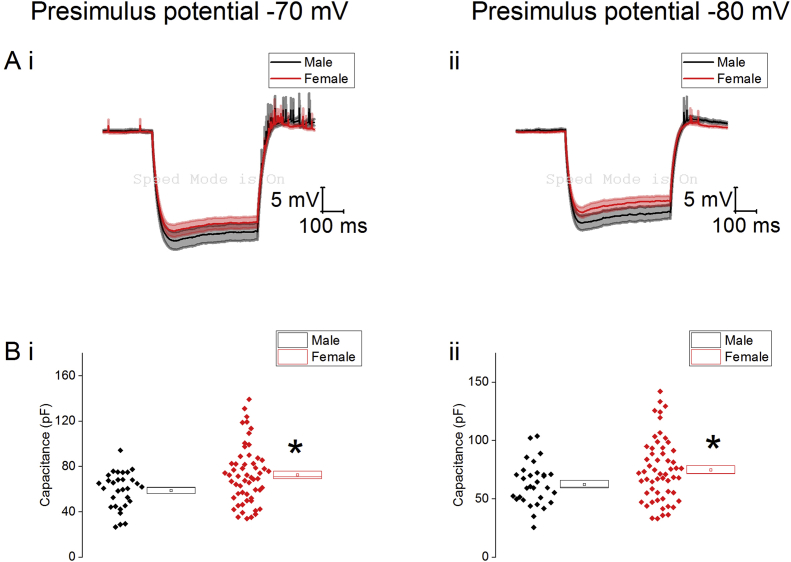
A) Average response to the injection of −40 pA of current from a prestimulus potential of −70 mV (i) and −80 mV (ii). B) Capacitance of Type II cells from a prestimulus potential of −70 mV (i) and −80 mV (ii), ∗ = p < 0.05.

We also examined the passive membrane properties of this cohort following the injection of −40 pA of current (averages shown in Fig. 4). From a prestimulus potential of −70 mV there was no significant effect of sex on input resistance (male: 527 ± 47 MΩ, n = 30, female: 473 ± 30 MΩ, n = 56, Mann-Whitney U, p = 0.21) however from a prestimulus potential of −80 mv the male cohort had a trend towards a higher input resistance which did not reach statistical significance (male: 430 ± 37 MΩ, n = 30, female: 371 ± 28, n = 56, Mann-Whitney U, p = 0.054). There was no effect of sex on sag or membrane time constant (Table 11). The capacitance was significantly higher in the female cohort (−70 mV: male: 59 ± 3 pF, n = 30, female: 73 ± 3 pF, n = 56. −80 mV: male: 63 ± 3 pF, n = 30, female: 75 ± 4 pF, n = 56, two-way ANOVA, p = 0.01).

**Table 11:** Passive membrane properties of Type I neurons. Mann-Whitney U (MW) statistical test, degrees of freedom in two way ANOVA = 1.

Parameter and prestimulus potential		Male	Female	P value	Statistical test
Tau (ms)	−70 mV	29 ± 2 (n = 30)	31 ± 1 (n = 56)	0.7	Two-way ANOVA
	−80 mV	25 ± 1 (n = 30)	25 ± 1 (n = 56)		
Sag (%)	−70 mV	12 ± 1 (n = 30)	12 ± 1 (n = 56)	0.3	Two-way ANOVA
	−80 mV	16 ± 2 (n = 30)	19 ± 1 (n = 56)		

**Fig. 5:**
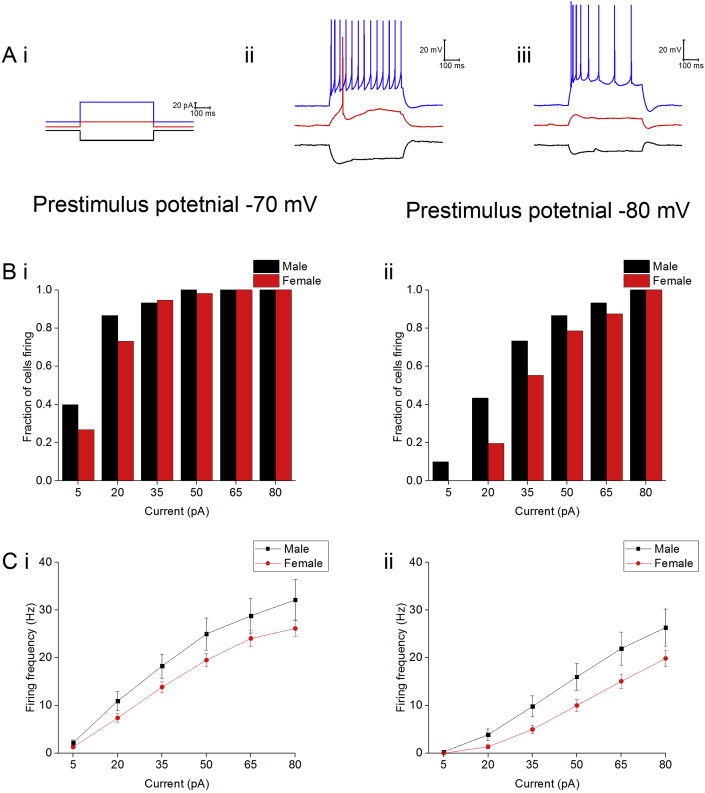
A) Sample traces showing the injection of −40 pA, +20 pA and +80 pA of current (i) and the responses from a (ii) male and (iii) female cell. B) The proportion of cells firing in response to a series of depolarising current injections from a prestimulus potential of −70 mV (i) and −80 mV (ii). C) Firing frequency in response to a series of depolarising current injections from a prestimulus potential of −70 mV (i) and −80 mV (ii), ∗ = p < 0.05.

Finally, we examined the effect of sex on excitability of Type II neurons. Excitability was characterised in a number of ways; these included determining the proportion of cells firing in response to a series of depolarising current injections, examining the firing frequencies during these current injections and measuring latency to the first spike following the injection of +80 pA of current (Fig. 5). From a prestimulus potential of −80 mV there was an increased likelihood of firing in the male cohort following the injection of the weakest current stimuli (5 pA and 20 pA); in response to the injection of 5 pA of current 3/30 of the males cells fired while 0/56 female cells fired (chi-squared, p = 0.02), in response to 20 pA of current 13/17 male cells fired while only 12/56 female cells fired (chi-squared, p = 0.03). There were no other significant differences in the proportion of cells firing following any other current injections or from a prestimulus potential of −70 mV. Cells in the male cohort fired at a higher frequency then those in the female cohort in response to depolarising stimuli (repeated measure three way ANOVA, p = 0.04). There was no effect of sex on spike latency from either prestimulus (−70 mV: male: 9 ± 1 ms, n = 30, female: 12 ± 1 ms, n = 56, Mann-Whitney U, p = 0.08. −80 mV: male: 15 ± 1 ms, n = 30, female: 24 ± 4 ms, n = 56, Mann-Whitney U, p = 0.10).

**Fig. 6:**
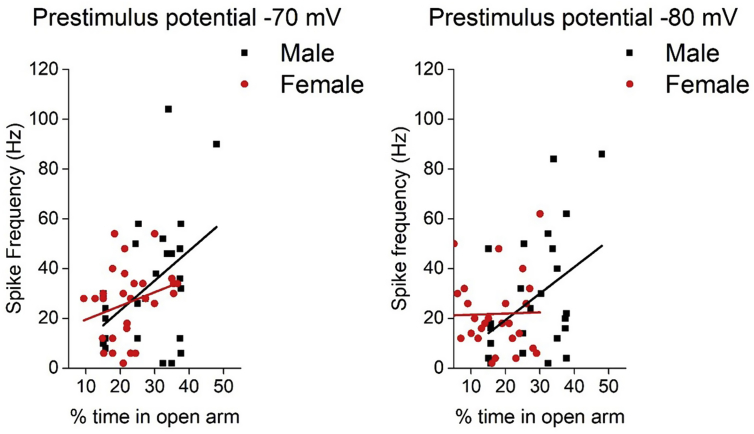
Correlation between firing frequency of Type II neurons and the percentage time spent in the open on the elevated zero maze. Male cohort examined 25 cells taken from 13 animals, female cohort examined 30 cells taken from 19 animals.

In order to assess the possible role of this population in behaviour, potential correlations between the electrophysiological properties of Type II neurons and behaviour on the elevated zero maze were examined. A strong correlation between firing frequencies following the injection of +80 pA of current and anxiety like behaviour was observed from both prestimulus potentials in the male cohort (−70 mV: Pearson correlation R-squared = 0.19, ANOVA, p = 0.03. −80 mV: R-squared = 0.17, ANOVA, p = 0.04, Fig. 6). This observation was not present in the female cohort (−70 mV: Pearson correlation R-squared = 0.08, ANOVA, p = 0.12. −80 mV: R-squared = 0.01, ANOVA, p = 0.88, Fig. 6).

## 4 Discussion

To our knowledge this is the first study to compare the electrophysiological properties of Type I and Type II neurons in the BNST^ALG^ in adult male and female mice aged 3–5 months. The main electrophysiological findings were a lower capacitance of Type I cells in the female cohort and a higher capacitance in the female cohort of Type II cells, which was paralleled by a lower intrinsic excitability. An additional aspect of this study was to consider the potential role of these physiologically distinct cell types in behaviour. In this respect, we found a negative correlation between time in the open component of the maze and the resting membrane potential of Type I neurons in males. We also found a positive correlation between the percentage time spend in the open arm and the firing frequencies of Type II neurons in males indicating a potential role for these populations in anxiety-like behaviour, once again this was only observed in the male population.

While a hypothesised a role for these different populations has been made based mainly on similar populations within the amygdala (Daniel and Rainnie, 2016), their functional roles have yet to be fully addressed. Type III cells in the BNST are CRF cells, they are highly responsive to stress and are thought to act as anxiety-inducing neurons (Dabrowska et al., 2013, 2016) however due to low n numbers in this study we were unable to identify sex specific changes in this population. Type I and Type II cells are hypothesised to be GABAergic and function as ‘anxiety off’ cells (Daniel and Rainnie, 2016). The correlation observed in male Type I neurons between resting membrane potential and time in the open arm could indicate an anxiety-inducing function in this unstressed model. The more hyperpolarising resting membrane potentials in the male cohort correlated with a decrease in anxiety. A more hyperpolarised resting membrane potential would indicate a decreased likelihood of firing as, all other things being equal, larger amounts of depolarising current would be required to bring the cell to threshold. To my knowledge there has been no papers which have identified specific neurochemical markers in this population therefore manipulation of this circuit in vivo would be difficult with our current level of understanding.

The correlation between behaviour and excitability of Type II cells supports the anxiety inhibiting hypothesis of this population with a greater excitability of male neurons, as measured by spiking rate for a 80 pA current injection, correlating with the higher percentage of time spent in the open component of the maze. A key finding in this study was a lower excitability of Type II neurons in females. Theoretically such a sex-associated change in excitability could result from a number of underlying sources such as alterations in baseline levels of stress hormones such as corticosterone, a negative shift in AP threshold, altered AHPs, or changes in input resistance. In the case of Type II neurons in the BNST^ALG^ the ∼15% higher input resistance observed in the male cohort is a likely contributing factor to the difference between the sexes. Although this difference in input resistance was not statistically significant in its own right, it may be enough to influence excitability, especially when combined pro-excitability differences in other parameters in females that also fail to reach significance in their own right, for example the slightly more negative AP threshold. What may be an important feature in the excitability of type II cells is the seemingly larger size of these neurons in females, as judged by a circa 20% larger calculated capacitance. With a uniform conductance per unit surface area of membrane this size difference would underpin the lower resistance of type II females BNST neurons. In theory the decrease in excitability of Type II neurons in the female cohort would lead to an increase in anxiety; however, there were no statistically significant sex-specific effects on percentage time spent in the open component of the elevated zero maze. One possible reason for this is the absence of data on Type III neurons. Type III neurons have been shown to play a key role in stress and anxiety (Dabrowska et al., 2016), therefore changes in Type III neurons, potentially masked by low statistical power, may have a more powerful effect then the alterations reported here, making the interpretation of the current data set more difficult.

The electrophysiological characterisation of neurons in the BNST at the cellular level, along with the examination of functionality of these populations, was originally based on studies performed with male rats (Hammack et al., 2007). This characterisation was recently expanded to other mammals including male mice and rhesus macaque; where a similar classification of cells appears to exist (Daniel et al., 2017) albeit in different proportions across the different species. In the present report we found a higher proportion of Type I and Type II cells with fewer recordings being made in Type III cells. This likely results from the majority of the recorded neurons in the current report being located in the undefined anterolateral area, an area where Type III neurons are sparser. An interesting addition to this work would be an extensive examination on the effect of sex on the CRF population (Type III cells) within the BNST however this falls outside the remit of the current report.

This is not the first study to examine the electrophysiological properties of female neurons located in the BNST^ALG^, Williams et al. (2018) found similar proportion of cell types to be present in the BNST^ALG^ of the female rat, while our group have previously found similar results in female mice of a similar age range (Smithers et al., 2017). The correlations observed in these studies were only significant in the male cohorts. This would lead one to question if such classifications are physiologically relevant in females. There are a number of possibilities why these classifications may alter between sexes, for example changes in I_h_ which would alter the sag of the cells, sag being one of the key parameters on which Type I and Type II cells are characterised. Equally changes in I_T_, which is believed to be responsible for rebound firing in Type II cells, may have led to the misclassification of proportions of this population. Reclassification of these cells in females may be required for an accurate representation of the roles of cells within the BNST. These discrepancies in classification highlights the problem of sex bias in our electrophysiological characterisation of populations, especially those in a sexually dimorphic brain regions such as the BNST.

Here we have provided insight into the differences in intrinsic properties of neurons located in the BNST^ALG^ between sexes in an unstressed model. These differences may provide a component of the physiological basis for changes in susceptibility to anxiety-related disorders. To our knowledge this is the first study to directly compare anxiety-like behaviour in relation to Type I and Type II cells in the BNST and has provided insight into the possible functionality of these different populations in the BNST.

## Declaration of conflict of interests

None.

## Funding

This research was funded by the University of Exeter and AstraZeneca.
